# Extreme Evolutionary Disparities Seen in Positive Selection across Seven Complex Diseases

**DOI:** 10.1371/journal.pone.0012236

**Published:** 2010-08-17

**Authors:** Erik Corona, Joel T. Dudley, Atul J. Butte

**Affiliations:** 1 Lucile Packard Children's Hospital, Stanford, California, United States of America; 2 Department of Pediatrics, Stanford University School of Medicine, Stanford, California, United States of America; University of Wisconsin, United States of America

## Abstract

Positive selection is known to occur when the environment that an organism inhabits is suddenly altered, as is the case across recent human history. Genome-wide association studies (GWASs) have successfully illuminated disease-associated variation. However, whether human evolution is heading towards or away from disease susceptibility in general remains an open question. The genetic-basis of common complex disease may partially be caused by positive selection events, which simultaneously increased fitness and susceptibility to disease. We analyze seven diseases studied by the Wellcome Trust Case Control Consortium to compare evidence for selection at every locus associated with disease. We take a large set of the most strongly associated SNPs in each GWA study in order to capture more hidden associations at the cost of introducing false positives into our analysis. We then search for signs of positive selection in this inclusive set of SNPs. There are striking differences between the seven studied diseases. We find alleles increasing susceptibility to Type 1 Diabetes (T1D), Rheumatoid Arthritis (RA), and Crohn's Disease (CD) underwent recent positive selection. There is more selection in alleles increasing, rather than decreasing, susceptibility to T1D. In the 80 SNPs most associated with T1D (p-value <7.01×10^−5^) showing strong signs of positive selection, 58 alleles associated with disease susceptibility show signs of positive selection, while only 22 associated with disease protection show signs of positive selection. Alleles increasing susceptibility to RA are under selection as well. In contrast, selection in SNPs associated with CD favors protective alleles. These results inform the current understanding of disease etiology, shed light on potential benefits associated with the genetic-basis of disease, and aid in the efforts to identify causal genetic factors underlying complex disease.

## Introduction

Humans have gone from existing solely in Africa to inhabiting every continent on Earth [1]. More recently, humans have begun cultivating specialized food-crop, domesticating animals, and living in towns and cities. Such environmental changes are known to alter common genetic variation via the positive selection of advantageous mutations [2]. As many populations were exposed to new food sources, diseases, and cultural lifestyles, positive selection likely played a major role in shaping the genetic architecture. A positive selection event represents a net gain of fitness, and there is room for the simultaneous selection of harmful mutations if they are linked to a relatively strongly beneficial mutation [3]. This can occur when a locus is in linkage disequilibrium (LD) with a beneficial mutation. Alternatively, the beneficial mutation may simultaneously harbor a harmful component [4]. Positive selection may occur as long as the benefits outweigh the harm. Therefore, increased susceptibility to disease may accompany a fitness-increasing mutation introduced by positive selection.

Complex diseases contain many distinct associations across the human genome that contribute only slightly to the absolute risk of disease [5]. These disease-associated mutations may undergo positive selection if they are simultaneously associated with relatively strongly beneficial traits. For example, the sickle cell mutation in the *Hemoglobin-B* (*HBB*) gene was found to be the target of positive selection due to its properties related to malaria resistance, despite its simultaneous role in introducing sickle cell disease [3]. It has also been recently shown that the variants in the antiviral response gene *IFIH1* associated with protection against enterovirus infection simultaneously increase susceptibility to Type 1 Diabetes (T1D) [6]. In this case, the benefits of having variants of the *IFIH1* gene that increase susceptibility to T1D depend on the prevalence of enterovirus infection. Having *IFIH1* gene variants increasing susceptibility to T1D may be considered advantageous and undergo positive selection if the probability of being exposed to the virus were high.

Rheumatoid Arthritis (RA) can be detected in human skeletal remains, and likely originated from the Americas and spread to Europe after the pre-Columbian era ended, possibly by a microorganism or allergen that is a necessary trigger for the disease [7]. This paves the way for selection to proceed strongly for potential benefits associated with the genetic-basis of RA. Prior to exposure to microorganisms or allergens required for the onset of RA, there would be no disadvantage to having mutations associated with RA in European populations. Ultimately, the cumulative contribution of such mutations with low effect sizes may play a large role in causing the disease.

It is currently unknown how much of the genetic-basis of complex disease originates from genetic mutations driven to prevalence by positive selection pressures. Blekhman *et al.* demonstrated that coding positions within disease associated genes underlying a number of complex human diseases are more rapidly evolving than coding regions of genes not associated with disease [8]. This suggests that evolutionary changes and natural selection may play a role in regions associated with complex disease.

We define a risk/susceptibility allele as the allele associated with more disease cases in the GWAS data used for our study, while the protective allele is necessarily present more often in healthy controls. Finding positively selected risk-associated alleles in complex human disease is challenging. It is easiest to learn more about the origins and history of disease when a mutation simultaneously confers a selective advantage while increasing susceptibility to disease in the same environment. It becomes more challenging when the risk-associated allele may falsely appear to be the target of selection if it is linked to a very advantageous allele due to linkage disequilibrium. To fully explain the origins of such risk-associated alleles, the nearby target of positive selection must first be identified.

An allele increasing susceptibility to disease in today's environment may have increased fitness only in an alternative environmental context [9], which makes it difficult to determine how (now absent) fitness-increasing properties can fully explain the history and origins of disease. While it is well established that more strongly deleterious mutations exhibit evolutionary profiles that differ from more neutral forms of variation in the human genome, it has been challenging to assess how much of a role natural selection has played in weakly deleterious mutations [10]. In previous studies excluding non-coding SNPs, it was shown that SNPs within complex disease associated genes are likely to be undergoing positive selection [11] [8], even in diseases having little impact on fitness [12]. If weakly deleterious mutations rise to prevalence via the positive selection of separate fitness increasing traits, we expect to find positive selection within relatively weakly deleterious mutations.

The first steps in addressing the issue of positive selection in complex diseases (including Crohn's Disease, Type 1, and Type 2 Diabetes) were taken in a study that scanned specific disease associated SNPs for positive selection [10]. This study examined an exclusive set of associated SNPs to avoid false associations, but excludes many hidden associations found in moderate association p-values. Consequently, while positive selection was found in individual SNPs, the search for positive selection of the overall genetic-basis of the each disease was inconclusive. A more thorough approach with positive selection detection methods proven to be more sensitive is warranted. Other approaches employing evolutionary analysis to characterize the genetic basis of disease are not applicable to complex disease. For example, selective pressures acting on specific conserved codon positions are used to predict deleterious mutations in human disease genes [13]. While evolutionary analysis of coding SNPs (cSNPs) within conserved amino acid sequences is informative most often for monogenic and Mendelian diseases, the same approach holds less utility in the analysis of complex, polygenic disorders due to association of many low-risk non-coding SNPs.

Modern approaches that incorporate haplotype structure have more power to detect recent positive selection [14], making them ideal for finding evidence of selection among complex diseases that have only recently emerged in the human genome. Detecting positive selection in loci associated with complex disease informs on the evolutionary history of disease and narrows down the search for positively selected components, possibly exposing the presence of unknown advantageous functions associated with the genetic basis of such diseases. These methods also indicate whether selection is acting on the major or minor allele.

It has been shown that different diseases can share association to the same SNPs, and that while one allele increases disease risk for one disease, the other allele may decrease disease risk for another [15]. By extending this principle, it is possible to determine whether selecting for protection against a disease also selects for increased risk for a separate disease.

Previous studies have focused on explaining the evolutionary role of the most highly selected genes in the human genome [14], [16], genes leading to monogenic disorders [17], or searching for selection within a handful of candidate genes [18], [19]. This study explores the role of selection across all SNPs moderately (p-value <0.05) associated with seven complex diseases characterized by the Wellcome Trust Case Control Consortium (WTCCC) within the context of population-based evolutionary histories [20]. In particular, this study investigates i) the relative patterns of positive selection across the WTCCC disease panel, ii) differences in positive selection signal strength in risk and protective alleles of disease-associated SNPs, and iii) proposes a method for identifying regions of interest likely to be strongly associated with disease despite modest association p-values from GWA studies. Insights into the selective pressures acting on disease-associated SNPs inferred from GWA studies offer a novel perspective that promises to augment the biological interpretation of the results and potentially serve as a complementary method for prioritizing disease-associated SNPs for follow-up validation studies. The overall aim of this study is to find evidence of positive selection within the genetic-basis of complex disease.

## Results

The Wellcome Trust Case Control Consortium (WTCCC) only identified twenty-four independent loci associated with the seven diseases at p <5×10^−7^; however, we expected there to be many additional associated loci likely hidden among SNPs with moderate p-values of association. Therefore, the association p-value threshold of 0.005 was used throughout this study in order to capture a larger proportion of these hidden associations, and any SNP with a p-value below this threshold is referred to as “associated”. We expect an increasing proportion of false associations as the p-value threshold is increased. Every allele of each associated SNP was evaluated for positive selection using both the integrated Haplotype Score (iHS) [14] and Long Range Haplotype (LRH) [21] methods, and was normalized with respect to the allele frequency and the ancestral or derived allele state (see Methods). Normalizing procedures are performed on all positive selection scores before any data analysis takes place. A different distribution of positive selection scores using both iHS and LRH is observed when the data is partitioned into distinct allele frequency scores as well as ancestral or derived allele states. This occurs because higher frequency alleles are older and their surrounding regions have had more time to undergo recombination, which makes it harder to detect positive selection. Likewise, ancestral alleles are older and have more recombination in the surrounding region. We also control for linkage disequilibrium by partitioning the entire genome into haplotype blocks and only including the SNP most strongly associated with disease in our analysis prior to any data analysis. This avoids the problem of measuring the same positive selection event twice (see Methods).

The number of SNPs moderately associated with each of the seven studied diseases is shown in Table 1. Out of the 1896 SNPs with moderate association with Type 1 Diabetes (T1D; p-values <0.005), there are 80 SNPs with strong evidence for selection, defined as having an absolute iHS score over 2.2. Calculating iHS values for each SNP yields two scores, one for each allele [14]. It is thus possible to further divide SNPs into those where selection is stronger for the allele increasing susceptibility to disease (i.e. the risk allele) and those in which selection favors the other “protective” allele. As shown in Table 1, we found surprising asymmetry in the selection of protective alleles compared to risk-associated alleles for certain diseases. We expect the same number of risk and protective alleles to be selected under the neutral model; in contrast to this, 58 alleles associated with increased risk of T1D exhibit selection, compared to 22 for protective alleles, revealing an asymmetrical distribution (binomial test p-value 7.01×10^−5^ against the null hypothesis of equal likelihood of risk and protective alleles to show stronger positive selection).

**Table 1:** Analysis of selection within risk-associated and protective alleles.

Disease	Number of SNPs associated with disease with p <0.005	Number of SNPs associated with disease with p <0.005 and |iHS| >2.2	Risk Allele Is More Selected	Protective Allele is More Selected	Binomial Test p-value
**T1D**	1896	80 (4.22%)	58	22	7.01×10^−5^
**T2D**	1632	42 (2.57%)	16	26	0.16
**CD**	1658	47 (2.83%)	13	34	3.09×10^−3^
**CAD**	1583	37 (2.34%)	21	16	0.51
**RA**	1695	48 (2.83%)	27	21	0.47
**HT**	1578	33 (2.09%)	17	16	1.00
**BD**	869	22 (2.53%)	4	18	4.34×10^−3^

Type 1 Diabetes risk alleles show significantly more selection than protective alleles, while Crohn's Disease and Bipolar Disorder show the opposite trend. We considered the set of moderately associated SNPs (p-value <0.005) across seven diseases studied by the Wellcome Trust Case Control Consortium. In Type 1 Diabetes, Crohn's Disease, and Bipolar Disorder, we see significant bias towards strong selection (defined as having an absolute iHS score >2.2) among these moderately associated SNPs. Each SNP represents a risk allele (allele increases susceptibility to disease) and a protective allele (the other allele). Among moderately selected SNPs associated with Type 1 Diabetes, 58 are SNPs in which the risk allele shows more selection, and only 22 are SNPs in which the protective allele shows more selection, showing that risk alleles are more likely to have undergone positive selection (p-value  = 7.01×10^−5^).

Crohn's Disease (CD) shares this asymmetrical distribution in the opposite direction, with 34 of 47 associated and positively selected SNPs exhibiting selection towards protective alleles (binomial test p-value 3.09×10^−3^). Bipolar Disorder (BD), like CD, also shows asymmetry in selecting for protective alleles, with 18 out of 22 alleles showing stronger selection for the protective allele. The findings in Table 1 were reproduced using the LRH test to detect positive selection yielding results matching very closely (see Table S1).

**Figure 1:**
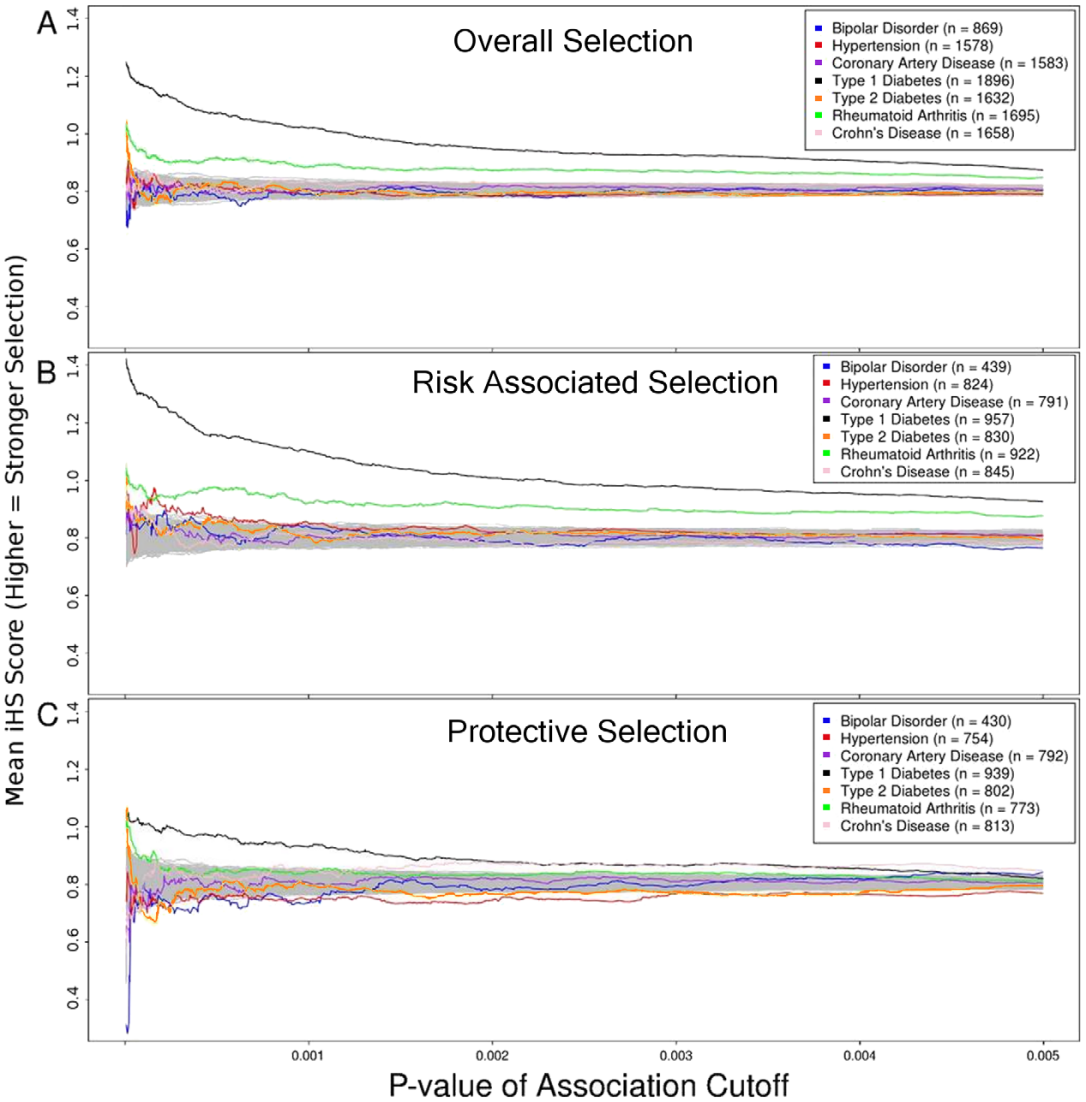
Selection in 7 WTCCC diseases. Comparison of selection pressures reveals stark heterogeneity across the 7 diseases studied. The x-axis represents the p-value of association cutoff used for each disease when calculating the mean iHS score (y-axis). Figure 1a shows Type 1 Diabetes and Rheumatoid Arthritis have strong evidence of positive selection. Type 2 Diabetes shows signs of positive selection only in the most strongly associated SNPs (left side of figure). Figures 1b and 1c expose differences in selection of risk-associated and protective alleles. Crohn's Disease shows stronger positive selection of protective alleles versus risk alleles. Like Type 1 Diabetes, Hypertension shows stronger selection for risk alleles. The gray regions represent a neutral random region used as a control, created by randomizing the data (see Methods).

Figure 1 shows the cumulative mean positive iHS selection score for SNPs at or stronger than threshold p-values of association, for all seven diseases. Interestingly, we find the strongest selected SNPs are found among the most significantly associated SNPs for T1D (Fig. 1a). Rheumatoid Arthritis (RA) also shows a concentration of high selection scores within the most significantly associated SNPs, as does Type 2 Diabetes.

The gray regions in Figure 1 represent neutral selection, made up of 1000 simulated control diseases having a random distribution of positive selection scores across SNPs with low p-values of association. In order to create each neutral control disease, all sets of SNPs from each of the 7 diseases were combined. From this combined set of SNPs, 86,972 SNPs were randomly drawn (matching the size of the data set for an LD controlled WTCCC disease). Each individual control disease then undergoes a random permutation of all of its positive selection scores, thereby producing a random distribution of positive selection scores in SNPs having low association p-values.

While Table 1 shows that three diseases show bias towards selection of risk-associated alleles or protective alleles, it does not show whether the selection itself is stronger among the risk associated alleles or protective alleles. Figures 1b and 1c show that the asymmetry of evidence for positive selection in risk and protective alleles shown in Table 1 is present within the most significantly associated SNPs.

Surprisingly, T1D shows the strongest selection pressure for its risk-associated alleles (Figure 1b), compared to the other six diseases and compared to its protective alleles (Figure 1c). RA and Type 2 Diabetes (T2D) also show strong selective pressure for both its risk-associated and protective alleles, but this selection is symmetrical, consistent with the results in Table 1. Table 1 shows that selection in CD more frequently acts on protective alleles; consistent with this, Figures 1b and 1c show that among protective alleles, there is a very strong signal of positive selection compared to risk-associated CD alleles (which show no deviation from the gray neutral region).

**Figure 2:**
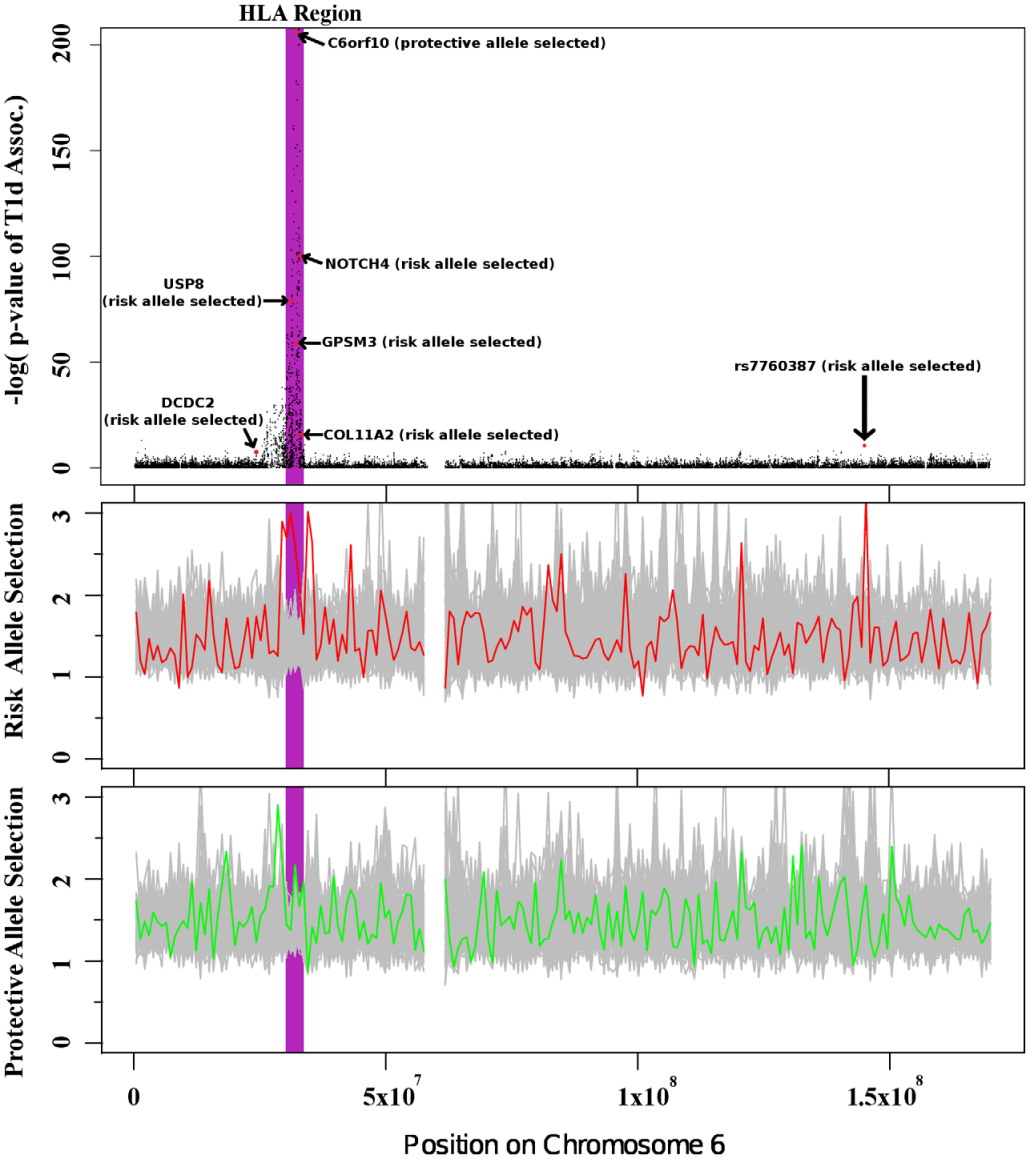
Selection of Type 1 Diabetes risk associated alleles in chromosome 6. Selection of associated alleles in chromosome six indicates that selection occurs more often on risk associated alleles versus protective alleles. The y-axis in the top plot represents how strongly each SNP is associated with Type 1 Diabetes. The y-axis in the bottom two plots represent how confident we can be that susceptibility alleles and protective alleles have been selected in each chromosomal region. The HLA (purple) region contains the strongest signs of positive selection for susceptibility alleles, yet the signal is within the neutral region for protective alleles. The second peak on the right side of the figure shows selection favors risk alleles in a region only moderately associated with the disease. Such asymmetry in selecting for risk-associated alleles suggests that SNPs in this region are more likely to be associated with the disease than the p-values suggest. Similar scans for regions moderately associated with diseases containing mutually exclusive selection of risk or protective disease alleles could be used to find novel associations. The gray regions in the bottom two graphs represent random neutral regions produced by randomizing the data (see Materials and Methods S1 for details).

Analyzing GWA studies in the context of selection can complement and augment the standard p-value of association and aid in the identification and characterization of causal loci for follow-up validation studies. Figure 2 places the SNPs on chromosome 6 associated with T1D within their evolutionary context. Deviations from the gray control region are observed in favor of susceptibility alleles in two regions, one in the HLA region and another towards the right side of the figure. T1D SNPs show more selection for risk than for protective alleles in the HLA region (left peak in Fig. 2). Another peak of selection is seen within a region with the positively selected SNP rs6917204 in linkage disequilibrium with the moderately T1D-associated SNP rs7760387. Many true associations are likely hidden among those SNPs with moderate p-values of association produced in GWA studies. Since selection is exclusively detectable in risk alleles within some moderately associated regions, it may be reasonable to up-weight the importance of these kinds of SNPs for follow-up deep sequencing and functional validation.

An inclusive list of all genes containing associated SNPs showing signs of recent positive selection is included in Table S2. In order to produce an inclusive list of associated SNPs that are likely undergoing positive selection, a p-value of association cutoff of 0.005 is maintained. In addition to meeting this threshold, all SNPs in Table S2 have an absolute iHS score above 1.645 *and* an LRH score below 0.05, representing SNPs that pass the top 95^th^ percentile for both iHS and LRH. Two missense mutations show up in this list for T1D, including *MOGS* (in SNP rs1063588), which encodes the first enzyme in the N-linked oligosaccharide processing pathway. A second SNP (rs1525791) within the *POU6F2* gene shows up for four different diseases: T1D, T2D, CD, and BD. The selected allele in this SNP represents the risk-associated allele for all four diseases. Table S3 shows all selected SNPs appearing in more than one disease.

## Discussion

Consistent with previous studies, we find positive selection is acting on loci associated with complex disease, when viewed from the perspective of Genome-Wide Association Studies (Fig. 1). While one might expect positive selection to eradicate risk-associated mutations and favor protective variants, like we find for CD (Table 1), we also surprisingly find the strongest selection working in favor of risk-associated alleles in both T1D and RA (Fig. 1b and Table 1).

It may be the case that some risk alleles are positively selected individually or as components of an abstract biological function due to a presently unknown benefit they confer on the host. This implies that there may be hidden benefits associated with some of the RA and T1D positively selected risk alleles. Positive selection in favor of risk alleles may occur if these alleles lead to an advantageous trait in an alternative environmental context (e.g. protection from pathogens). It is worth mentioning that there must be some reason why the ancestral allele characterized ancient populations in the first place in the cases where the derived allele shows strong signs of positive selection. There are many possible explanations for this. Possibilities include genetic drift driving a benign mutation to fixation in ancient populations, only to have this benign mutation become deleterious relative to a different allele in a modern environment. Another possibility is that an ancient environment may have also caused the ancestral allele to undergo positive selection, only for a modern environment to induce the ancestral allele to undergo negative selection.

There is recent evidence suggesting that protection from pathogens helps explains why positive selection has occurred for T1D susceptibility alleles. Rare variants in the antiviral response gene *IFIH1* have recently been shown to be protective against T1D [6]. The protective alleles are functionally deleterious to *IFIH1*, effectively reducing the ability to mediate an immune response against enterovirus infection. Recent immunological studies of beta cells in patients recently diagnosed with T1D have shown an abundance of enteroviral capsid proteins in the islet cells of affected patients, whereas the protein is found to be scarce among the beta cells of healthy controls [22]. It is plausible that signatures of recent positive selection in T1D and other autoimmune diseases are due to an overactive immune system driven at least in part by an adaptive immune response to viruses. Due to the evolutionary trajectory of T1D favoring susceptibility alleles and the severe effect on fitness in afflicted individuals, we would expect that this evolutionary event would have happened recently in human evolution. While T1D is rapidly fatal without insulin therapy, there was likely a net selective pressure favoring intense immune responses to enterovirus, even with T1D as an occasional consequence.

RA has been found to originate from Native American populations from the Green River region in west central Kentucky. There are verified cases of RA in this population as far back as 6,500 years ago. No signs of RA were found in 63 archaeological sites bordering the original area in central Kentucky, where it was originally found [7]. Yet, there is documented spread in America over time. The first evidence of RA outside the original “catchment” area occurs in western Ohio about 1,100 to 800 years ago. At the same time, virtually no incidence of RA in other parts of the world has been found towards the end of the pre-Columbian era in 1785. This suggests that some environmental factor, perhaps a microorganism or allergen, might play a critical role in the cause of RA [7]. Our analysis reveals that there is a huge disparity in positive selection scores between alleles increasing and decreasing susceptibility to RA. Susceptibility alleles show very strong signs of positive selection, while alleles decreasing susceptibility are nearly devoid of any signs of positive selection (Fig. 1b–1c). The history of RA helps explain why susceptibility alleles show signs of positive selection in European-derived populations (Fig. 1). Since RA was non-existent in these populations during the pre-Columbian era, there were probably no disadvantages to selecting for the genetic-basis of RA. Indeed, there may have been many benefits associated with selecting for RA susceptibility alleles. Tuberculosis is responsible for millions of deaths worldwide in recent human history, with one in four deaths caused by tuberculosis in Western Europe in the 19^th^ century alone. It is suspected that this disease has historically acted as a powerful selective force. There is a stark correlation between populations having higher incidence of tuberculosis also having lower incidence of RA, and vice versa. It has been speculated that genetic variants enhancing resistance to tuberculosis underwent positive selection and provide the genetic basis for RA susceptibility today [9]. Our analysis is completely compatible with this theory, since we produce evidence that RA susceptibility alleles have undergone positive selection. In addition, tumor necrosis factor inhibitors alleviate symptoms of RA while simultaneously increasing the risk of infection from tuberculosis, *Myobacterium marinum tenosynovitis*, fungal infection, and other opportunistic infections [23], [24], [25], [26], [27]. It is clear that factors increasing susceptibility to RA also decrease susceptibility to infectious disease. RA and T1D are known to share associated variants [28], [29]. This may partially explain why there is a small, but detectable signal of positive selection in alleles decreasing susceptibility for RA. The evolutionary history of RA is unique in that a precise date of introduction of RA into European-derived populations has been established. We have shown that strong positive selection of RA susceptibility alleles is observed, most likely due to altered ability to fight infectious disease without increasing the risk of RA itself until the pre-Columbian era ended.

Not much is known about the history of Crohn's Disease (CD) as it does not leave unambiguous signs in skeletal remains, as is the case with RA. It is known that the incidence of CD increased during the 19^th^ century in industrialized countries. The rate of CD increases as under-developed countries become more industrialized (e.g. Japan and Brazil) [30]. Many bacteria are implicated in CD, including anaerobic organisms, paratuberculosis, Boeck's sarcoid, and mycobacteria. *Mycobacterial paratuberculosis* infection of the terminal ileum in cattle (Johne's disease) resembles also closely resembles Crohn's disease, which has suggested possible bacterial associations with CD [30]. Unlike RA and Type 1 Diabetes, CD shows more positive selection for alleles decreasing susceptibility to disease than for those increasing susceptibility. It may be the case that CD is in fact an ancient disease, the incidence of which was reduced due to natural selection against CD, only to see resurgence due to the advent of modern environments. However, many other possible scenarios could explain our findings, including shared genetic variants with a disease or trait that has undergone negative selection. CD is unique in the sense that while selection is detected as in RA and T1D, alleles decreasing susceptibility for CD are under positive selection, indicating a very different evolutionary history.

We acknowledge several limitations in our analysis. Controlling for LD by selecting only one SNP in each haplotype block after partitioning the genome may have complications in some regions of the genome. Haplotype blocks intuitively capture LD, but lack of complete haplotype block coverage (the fraction of the genome that is found neatly within haplotype blocks) complicates this approach [31]. More complex methods to control for LD will be considered for future works requiring a similar analysis. Another complicating issue is that both iHS and LRH belong to the same class of analytical methods for detecting selection, and it is not surprising that they indicate similar results. Yet, it has been shown that these two methods are in some ways complementary as they are better at detecting selected SNPs at different allele frequencies [16]. Overall, the results under iHS match the results produced with LRH with some changes in the magnitude of selection pressures on some diseases leading to more diseases appearing in the random neutral region in Fig 1 versus Figure S1 (more details on limitations in Materials and Methods S1). We acknowledge that it is unknown whether or not the most associated SNPs are causative; leading to confusion when we discuss selection for risk-associated alleles as the causal SNP may show the opposite selection pattern, that is, stronger selection of the protective allele. While this is certainly a possibility, it is unlikely to occur often. If a SNP has a very low p-value of association to a disease due to its proximity to the causative allele, it implies strong LD between the two SNPs. Due to strong LD, the risk-associated allele between the non-causative and the causative SNPs are more likely to be on the same haplotype block, making the risk-associated allele in a SNP in strong LD with the causative SNP an appropriate proxy. In addition, it should be noted that all discussion on the reasons for positive selection acting on these diseases necessarily remains speculation.

In summary, we observed stark heterogeneity in the overall patterns of positive selection across seven diseases. We find that the SNPs associated with T1D, RA, and CD show strong signs of positive selection. We also find that positive selection favors risk-associated alleles in T1D and protective alleles in CD, which is indicative of an evolutionary trajectory towards increasing and decreasing risk, respectively. In addition, we have demonstrated that selection analyses of GWAS results can complement and augment the basic p-value of association attributes (Fig. 2) as many regions appear to exclusively favor selection of risk or protective alleles.

## Methods

In EHH (extended haplotype homozygosity) based positive selection methods, there are two selection scores for each SNP (one for each allele). Both iHS and LRH are based on the EHH calculation. These two methods, and variants thereof have been applied to uncover signals of positive selection within genes related to susceptibility and resistance to infectious disease [16], innate and adaptive immunity [32], LDL cholesterol levels [33], and autoimmune disorders [34]. This method exploits the principle that alleles in relatively larger and over-represented haplotype blocks imply positive selection. These two methods produce biased scores depending on the allele frequency and ancestral/derived allele state. These limitations were overcome by performing Z-score normalization and rank normalization of each iHS and LRH measurement, respectively. Each allele of each SNP was grouped with other alleles having the same allele frequency in addition to having the same ancestral/derived state prior to performing Z-score (iHS) and inverse rank (LRH) normalization. All references to iHS and LRH score reference their normalized values.

**Figure 3:**
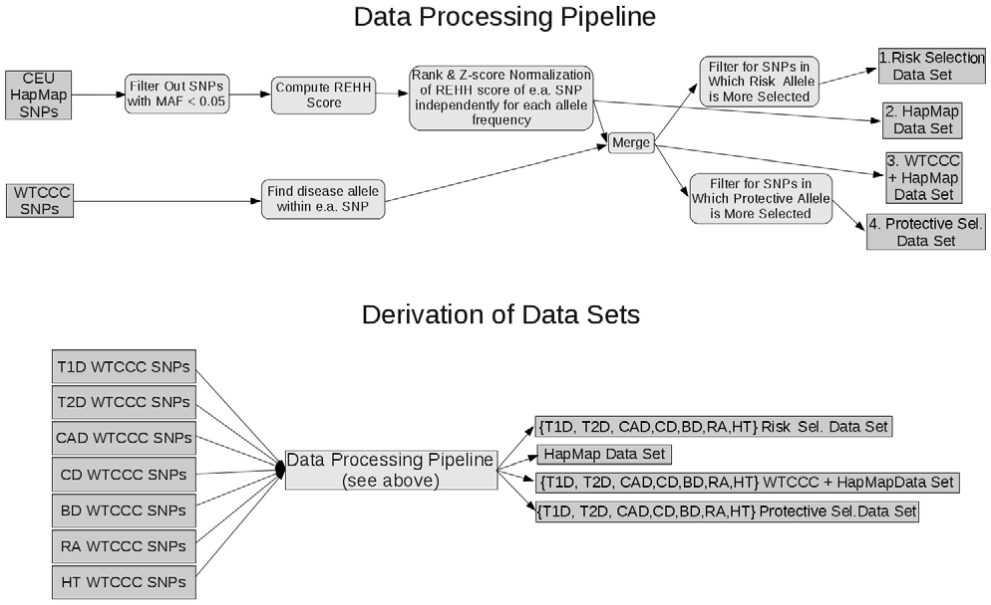
Project pipeline. The HapMap and WTCCC data sets are combined and partitioned to derive the data sets used for this study. First, the HapMap data set is filtered (SNPs with MAF <0.05 are excluded). iHS and LRH scores are then calculated for each SNP, which are Z-score and inverse rank normalized with respect to allele frequency and ancestral/derived allele state. The data is then merged with a WTCCC disease SNP data set after the “risk” allele has been extracted from the WTCCC SNP data set. This leads to 4 distinct SNP datasets; i) scored CEU HapMap SNPs, ii) the intersection of WTCCC SNPs and HapMap SNPs, iii) SNPs in which the susceptibility allele shows more selection iv) SNPs in which the protective allele show more selection. This data processing pipeline is used on all 7 WTCCC diseases, resulting in distinct data sets, which are then probed for disparities in positive selection.

**Figure 4:**
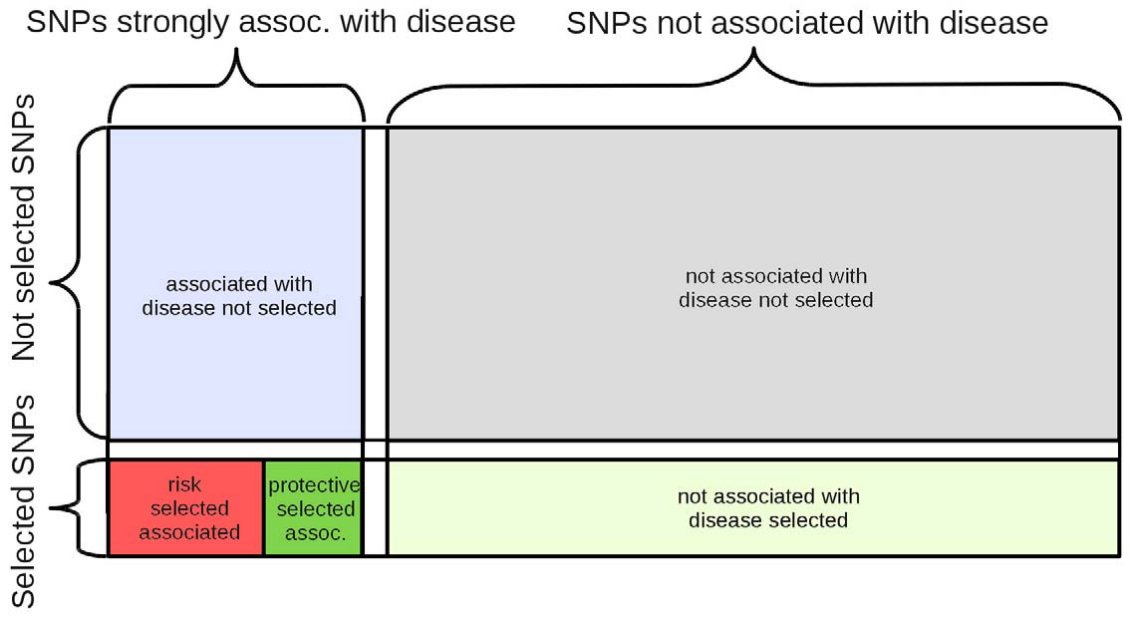
Partitioning the data set. The entire set of SNPs present in both the WTCCC and HapMap data sets was partitioned into five different categories. This study emphasizes associated SNPs partitioned into risk-associated selection and protective selected SNPs. The proportion of SNPs in these categories at cutoffs for selection and association (|iHS| >2.2 and rank normalized LRH score <0.01) is explored and used to test for differences in selection pressures among associated SNPs (p-value <0.005). Within associated SNPs, selection pressures between risk-associated selection (SNPs in which stronger selection is observed for the risk-associated allele), and protective selection are explored.

The WTCCC study used the Affymetrix GeneChip 500K Array set to conduct a GWA study made up of two European cohorts for each disease. These include 2000 affected individuals for each of the seven diseases as well as a common control group of 3000 individuals. This study exploits the existence of hidden associations by including moderately associated SNPs. While this will undoubtedly capture hidden associations, diminishing returns of these “hidden associations” are expected as one increases the p-value (Fig. 1). The entire project pipeline is shown in Figure 3. We took all associated SNPs from the 7 diseases studied by the WTCCC using high-density assays [20] and used both iHS and LRH to probe for evidence of positive selection in these SNPs using haplotype information from the population having European ancestry in Phase 2 of the International HapMap project [35]. For every SNP showing association with disease (p-value <0.005) that has recently undergone positive selection (|iHS| >2.2), we determined whether the selected allele within the SNP is associated with susceptibility to disease versus protection from disease (Table 1 and Table S1). Figure 4 shows how risk versus protective SNPs are partitioned in this study.

In every case, the positive selection score assigned to a SNP is simply the score from the major and minor alleles showing the strongest signs of positive selection. As mentioned, there are two selection scores for each SNP, one for each allele. This makes it possible to investigate positive selection of a disease's risk-associated alleles separately from its protective alleles. In addition to controlling for allele frequency and for the ancestral/derived allele state, it is important to take into consideration whether or not a disease appears to be exhibiting positive selection due to a few high-scoring non-independently associated regions in Linkage Disequilibrium (LD). This analysis relies on the independence of each positive selection score measurement. In order to overcome any potential issues with the non-independence of iHS and LRH calculations, the entire genome was partitioned into haplotype blocks using HapBlock software [36]. Haplotype blocks can be defined as regions of high |D'| or low haplotype diversity. The “Haplotype Diversity” method is used for partitioning the data into haplotype blocks [37] (more details provided in Materials and Methods S1). For each haplotype block, only the SNP most strongly associated with each of the 7 diseases (leading to a different data set for each disease) is chosen for this study, along with its corresponding iHS and LRH score (see Materials and Methods S1).

Randomized WTCCC and HapMap data was produced to detect deviations from neutral selection in Fig. 1 and Fig. S1. Each SNP comprising the 1000 neutral diseases (along with its positive selection score) are picked at random from each of the 7 diseases until the number of SNPs in each disease matched the number of SNPs in a WTCCC disease. The entire complement of the WTCCC disease panel resulting after the normalization and filtering procedures discussed was used to compute each random disease. Following this random selection of SNPs, the entire combined list of SNPs from the 7 diseases was randomly reassigned a positive selection score from this same combined list of SNPs, thereby creating 1000 “random neutral diseases” that have a random distribution of selection scores in associated SNPs. Figure 1 and Fig. 2 contain random neutral regions produced with this set of 1000 random diseases (see Materials and Methods S1 for more details).

Data for the seven diseases studied was obtained from the WTCCC1 data set [20]. WTCCC SNPs were intersected with CEU (Utah population) HapMap Phase II data (release 23a; only including autosomes). With the WTCCC data set, it is possible to deduce which allele within an associated SNP is increasing susceptibility to disease by looking at the genotype of the control group as well as those affected by the disease. Since LRH and iHS give scores for each allele of every SNP, we use the associated allele for each disease (derived from the WTCCC data set) and each allele's LRH and iHS scores (computed from the HapMap data set) in order to assess whether the evolutionary trajectory is towards increased or decreased susceptibility to the diseases studied. The HapMap data adds frequency and haplotype data to each SNP included in the WTCCC study. Intersecting the HapMap and WTCCC data sets yielded 352,191 Type 1 Diabetes, 352,202 Type 2 Diabetes, 352,199 Rheumatoid Arthritis, 352,200 Hypertension, 352,198 Crohn's Disease, 352,198 Coronary Artery Disease, and 332,576 Bipolar Disorder SNPs. These SNPs were then normalized and filtered to control for LD effects. Finally, they are partitioned as shown in Fig. 4. Emphasis is placed on SNPs associated with each disease (defined as p-value <0.005 for this study). Among these, positively selected SNPs were identified (iHS >2.2 and normalized LRH score <0.01). Such positively selected SNPs were tested to see if the risk and/or protective alleles have recently undergone positive selection. Ancestral and derived alleles were determined by downloading DBSNP [38], which uses a method that derives the ancestral allele by comparing human DNA to chimpanzee DNA [39]. This study makes use of data generated by the Wellcome Trust Case Control Consortium. A full list of the investigators who contributed to the generation of the data is available from www.wtccc.org.uk. Funding for the Wellcome Trust Case Control Consortium was provided by the Wellcome Trust under award 076113. Version WTCCC1 of the dataset was used for this study [20].

## Supporting Information

Materials and Methods S1 Materials and methods explaining limitations of this study, replication of the main results using LRH, and a list of SNPs associated with more than one disease showing some signs of selection.
(0.04 MB DOC)

Figure S1 Comparison of selection pressures reveals differences across the 7 diseases studied. The x-axis represents the p-value of association cutoff used for each disease when calculating the mean rank normalized LRH score (y-axis). Type 1 Diabetes shows extremely strong signs of positive selection. Crohn's Disease, Rheumatoid Arthritis, and Hypertension also exhibit evidence of positive selection. Figures S1b-S1c expose differences in the magnitude of selection strength. Crohn's Disease shows stronger positive selection of protective alleles versus susceptibility alleles. Hypertension shows positive selection almost exclusively for risk alleles. The gray regions represent a neutral random region used as a control which was made by randomizing the data (see Methods).
(1.95 MB TIF)

Table S1 Alleles of SNPs associated with disease (p-value <0.005) can either be SNPs in which the susceptibility allele shows more selection than the protective allele (risk SNPs) or SNPs in which the protective allele shows more selection (protective SNPs). When the intersection of Type 1 Diabetes associated SNPs (p-value 0.005) and strongly selected SNPs (LRH <0.01) are considered, 23 are SNPs in which the risk allele shows more selection than the protective allele, and only 8 are SNPs in which the protective allele shows more selection. This shows that risk alleles are more likely to have undergone positive selection (p-value  = 0.01).
(0.04 MB DOC)

Table S2 In order to produce an inclusive list of all disease associated SNPs that have recently undergone positive selection, all associated SNPs (p-value <0.005) having both a rank normalized LRH score below 0.05 and an iHS score greater than 1.645 (representing the 95th percentile in both LRH and iHS) are shown. SNPs in linkage disequilibrium with the SNPs appearing in these tables were not listed.
(0.16 MB DOC)

Table S3 Five of the SNPs listed in Table S2 appear in more than one disease. In particular, rs1525791 appears in four of the seven WTCCC diseases and the risk-associated allele in this SNP shows more selection than the protective allele. This is in contrast to rs204989, where the susceptibility allele for Type 1 Diabetes and the protective allele for Rheumatoid Arthritis are under selection.
(0.04 MB DOC)
