# Supplementary material for: Extreme Evolutionary Disparities Seen in Positive Selection across Seven Complex Diseases
Source: PLoS One. 2010 Aug 17;5(8):e12236. doi: 10.1371/journal.pone.0012236 (PMC2923198; doi:10.1371/journal.pone.0012236)
Supplement: Materials and Methods S1 — Materials and methods explaining limitations of this study, replication of the main results using LRH, and a list of SNPs associated with more than one disease showing some signs of selection. (0.04 MB DOC) [file pone.0012236.s001.doc]

**Materials and Methods S1: Extreme Evolutionary Disparities Seen in**

**Pos­itive Selection Across Seven Complex Diseases**

**Study Limitations**

It is important to acknowledge that the data is limited only to the SNPs that were found to be associated with the diseases studied in the WTCCC data set. If SNPs known to be associated with any of these diseases did not receive an association p-value below 0.005 in the WTCCC data set, they were excluded from the main results of this study. It is also important to point out that our results represent overall trends. Even if a few alleles for genes associated with insulin resistance have iHS and LRH scores indicative of selection, it will not necessarily show Type 2 Diabetes as a whole as having recently undergone positive selection. The cumulative signals of positive selection of every SNP strongly associated with each disease are considered. This leads us to conclude that if a disease is observed to fall within the control region (gray region in Fig. 1), a few specific elements of the disease may still be under strong positive selection, and therefore do not make the claim that a disease in the gray neutral region of Figure 1 is devoid of signatures of positive selection. We also acknowledge that WTCCC and other GWAS are using tagSNPs, which may not be causal SNPs. As these tagSNPs are thought to represent loci of importance, and are equally likely a priori to be causal, the positive selection differences we see across the seven diseases and between susceptibility and protective alleles represent true selection patterns associated with the genetic-basis of each disease.

**Details on Figure 1 and Figure S1** Figure 1 is made up of iHS values, which are in essence Z-scores. Figure 1a reports on selection of all associated SNPs as opposed to selection of a particular allele (risk or protective). All iHS values between major and minor alleles share symmetry in that the iHS value of the major allele is the same as the negative of the iHS value of the minor allele. Therefore, the distribution of iHS values for all SNPs in Figure 1a is the absolute value of all iHS values of each SNP’s minor allele as overall selection is intended to be captured. This explains why the randomized gray neutral region is not centered at an iHS value of zero, despite being a random distribution of Z-scores. In actuality, the gray neutral region represents a distribution of the absolute value of a set of Z-scores. This also holds true for Figure 1b and 1c. In Figure 1b, the data is comprised of SNPs in which the most selected allele is the susceptibility allele. This is in contrast to Figure 1c, where the protective allele shows more selection than the susceptibility allele.

Figure S1a shows overall selection of SNPs using the LRH score. In order to show overall selection of each SNP as opposed to selection of a particular allele, the LRH score (out of the LRH scores for the major and minor alleles) showing the strongest signs of selection is chosen as the overall positive selection score for each SNP. Since plotting the minimum LRH score out of the two alleles in each SNP causes the mean LRH score to be below 0.5 in Fig. S1a, the data is rank normalized a second time. This ensures that the mean is 0.5, causing the neutral region to be centered at an LRH score of 0.5. This is done to make Fig S1a have the same y-axis range as in Figures S1b-S1c. SNPs in which the risk-associated allele shows more selection than the protective-allele (using LRH scores) are used to produce Fig. S1b. Likewise, SNPs in which the protective-allele shows more selection than the risk-associated allele are used to Figure S1c.

**Supplemental Methods**

Both iHS and LRH scores are normalized after being grouped with all other SNPs having the same allele frequency and ancestral/derived allele states in order to enable the direct comparison of positive selection pressures on SNPs across all frequencies and ancestral/derived allele states. The inverse rank of each LRH score was taken, leading to LRH scores ranging from 0 to 1. The LRH score thus represents the fraction of SNPs with an identical SNP allele frequency having a larger LRH score, meaning the lower the normalized LRH score, the stronger the signal for positive selection. For example, a normalized LRH score of 0.01 indicates that only 1% of all alleles with the same frequency and ancestral/derived allele state, have a stronger positive selection signal.

Scores representing selection are iHS values in the top scoring 99^th^ percentile of all iHS scores. Since iHS values are in essence Z-scores, this corresponds to an iHS value of 2.2. Any SNP in which the minor or major allele shows an iHS value above 2.2 is taken as showing signs of selection in this study. While there are undoubtedly false positives at a cutoff of 2.2, there are obvious signs that this cutoff detects unambiguous signals of positive selection. For example, Table S2 shows that risk-associated alleles in SNPs associated with Type 1 Diabetes are more likely to be undergoing positive selection compared to protective alleles. Likewise, the top 99^th^ percentile of all LRH scores are chosen to represent SNPs under selection when analysis is performed using the LRH method. Since LRH is inverse rank normalized, any SNP with an LRH score below 0.01 is considered positively selected for the purposes of this study.

The two positive selection methods iHS and LRH do not always agree on whether the risk-associated allele shows more selection than the protective allele. The data was thus partitioned with respect to risk and protective allele positive selection twice, once for iHS and then again for LRH, resulting in a doubling of the data set.

**Figure 2 Details**

In Fig. 2, the chromosome is split into 200 regions. The iHS values are transformed by taking the negative log of the p-value associated with each iHS value (iHS values represent Z-scores). For each of the 200 bins, the median transformed iHS value is plotted along the chromosome in the bottom two plots to produce this broad representation of selection.

**Partitioning the data into haplotype blocks**

The software called “HapBlock” is used in partitioning the data [1]. The block partition with the minimum number of tag SNPs was chosen for this study, where tagSNPs are defined as SNPs that distinguish all common haplotypes. The Haplotype Diversity method is used for partitioning this data into haplotype blocks [2]. The threshold used to determine if a segment of haplotype is a block is 0.80. The threshold for common haplotypes in block partitioning is set to 0.07. The threshold to define common haplotypes in tag SNP selection is 0.80.

**References**

1. Zhang K, Qin Z, Chen T, Liu JS, Waterman MS, et al. (2005) HapBlock: haplotype block partitioning and tag SNP selection software using a set of dynamic programming algorithms. Bioinformatics 21: 131-134.

2. Patil N, Berno AJ, Hinds DA, Barrett WA, Doshi JM, et al. (2001) Blocks of limited haplotype diversity revealed by high-resolution scanning of human chromosome 21. Science 294: 1719-1723.
