# Supplementary figures and images for: Extreme Evolutionary Disparities Seen in Positive Selection across Seven Complex Diseases
Source: PLoS One. 2010 Aug 17;5(8):e12236. doi: 10.1371/journal.pone.0012236 (PMC2923198; doi:10.1371/journal.pone.0012236)

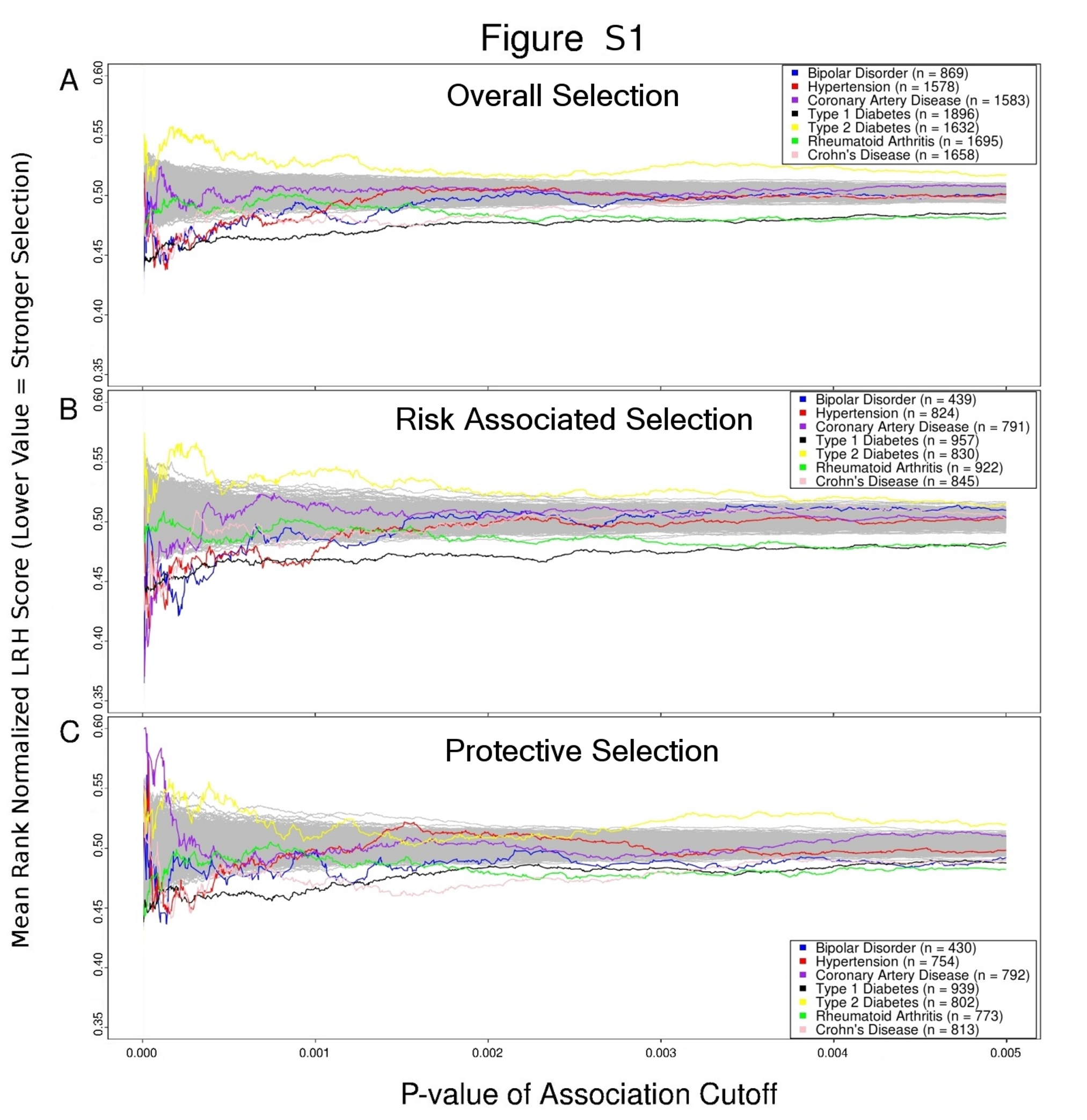

Supplement: Figure S1 — Comparison of selection pressures reveals differences across the 7 diseases studied. The x-axis represents the p-value of association cutoff used for each disease when calculating the mean rank normalized LRH score (y-axis). Type 1 Diabetes shows extremely strong signs of positive selection. Crohn's Disease, Rheumatoid Arthritis, and Hypertension also exhibit evidence of positive selection. Figures S1b-S1c expose differences in the magnitude of selection strength. Crohn's Disease shows stronger positive selection of protective alleles versus susceptibility alleles. Hypertension shows positive selection almost exclusively for risk alleles. The gray regions represent a neutral random region used as a control which was made by randomizing the data (see Methods). (1.95 MB TIF) [file pone.0012236.s002.tif]
