# Supplementary material for: Extreme Evolutionary Disparities Seen in Positive Selection across Seven Complex Diseases
Source: PLoS One. 2010 Aug 17;5(8):e12236. doi: 10.1371/journal.pone.0012236 (PMC2923198; doi:10.1371/journal.pone.0012236)
Supplement: Table S1 — Alleles of SNPs associated with disease (p-value <0.005) can either be SNPs in which the susceptibility allele shows more selection than the protective allele (risk SNPs) or SNPs in which the protective allele shows more selection (protective SNPs). When the intersection of Type 1 Diabetes associated SNPs (p-value 0.005) and strongly selected SNPs (LRH <0.01) are considered, 23 are SNPs in which the risk allele shows more selection than the protective allele, and only 8 are SNPs in which the protective allele shows more selection. This shows that risk alleles are more likely to have undergone positive selection (p-value = 0.01). (0.04 MB DOC) [file pone.0012236.s003.doc]

| **Disease** | **Number of SNPs associated with disease with p < 0.005** | **Number of SNPs associated with disease with p < 0.005 and LRH < 0.01** | **Risk Allele**  **Is More**  **Selected** | **Protective Allele is**  **More Selected** | **Binomial Test p‑value** |
| --- | --- | --- | --- | --- | --- |
| **T1D** | 1896 | 31 (1.64%) | 23 | 8 | 0.01 |
| **T2D** | 1632 | 13 (0.80%) | 4 | 9 | 0.27 |
| **CD** | 1658 | 20 (1.21%) | 6 | 14 | 0.16 |
| **CAD** | 1583 | 7 (0.44%) | 4 | 3 | 1.00 |
| **RA** | 1695 | 21 (1.23%) | 15 | 6 | 0.08 |
| **HT** | 1578 | 14 | 7 | 7 | 1.00 |
| **BD** | 869 | 8 (0.92%) | 5 | 3 | 0.73 |

Table S1
