# Supplementary material for: Extreme Evolutionary Disparities Seen in Positive Selection across Seven Complex Diseases
Source: PLoS One. 2010 Aug 17;5(8):e12236. doi: 10.1371/journal.pone.0012236 (PMC2923198; doi:10.1371/journal.pone.0012236)
Supplement: Table S2 — In order to produce an inclusive list of all disease associated SNPs that have recently undergone positive selection, all associated SNPs (p-value <0.005) having both a rank normalized LRH score below 0.05 and an iHS score greater than 1.645 (representing the 95th percentile in both LRH and iHS) are shown. SNPs in linkage disequilibrium with the SNPs appearing in these tables were not listed. (0.16 MB DOC) [file pone.0012236.s004.doc]

**Table S2**

**Table S2a – Type 1 Diabetes**

| **SNP** | **Chr** | **Chr Pos** | **Association P-value** | **iHS** | **LRH** | **Gene Symbol** | **Mutation** | **Allele Selected** |
| --- | --- | --- | --- | --- | --- | --- | --- | --- |
| rs3129934 | 6 | 32444165 | 1.56E-90 | 2.195348 | 0.033862751 | C6orf10 | Intron | Protective |
| rs2071278 | 6 | 32273422 | 9.14E-45 | 2.641524 | 0.007092058 | NOTCH4 | Intron | Risk |
| rs2524067 | 6 | 31353800 | 5.04E-35 | 3.503046 | 0.029488438 | USP8 | missense | Risk |
| rs3129818 | 6 | 30450945 | 1.91E-27 | 3.33904 | 0.003166597 | - | - | Risk |
| rs3132625 | 6 | 30455699 | 2.13E-26 | 3.436167 | 0.000392136 | - | - | Risk |
| rs204989 | 6 | 32269830 | 3.20E-26 | 2.212887 | 0.037739195 | GPSM3 | Intron | Risk |
| rs3130641 | 6 | 30872060 | 1.40E-21 | 2.377165 | 0.000799926 | - | - | Risk |
| rs3131050 | 6 | 30868004 | 4.67E-21 | 2.922976 | 0.000351074 | - | - | Risk |
| rs3132605 | 6 | 30847951 | 3.34E-20 | 2.533482 | 0.001668886 | - | - | Risk |
| rs406511 | 6 | 29484364 | 7.23E-15 | 3.096782 | 0.003721438 | - | - | Risk |
| rs376877 | 6 | 33132584 | 3.02E-14 | 2.141085 | 0.038831683 | - | - | Risk |
| rs1233491 | 6 | 29569709 | 1.36E-13 | 3.26203 | 0.002579934 | - | - | Risk |
| rs3117425 | 6 | 29368410 | 1.49E-12 | 1.995825 | 0.008884898 | - | - | Risk |
| rs1525791 | 7 | 39123083 | 2.63E-11 | 1.911989 | 0.00327387 | POU6F2 | Intron | Risk |
| rs2523742 | 6 | 30312378 | 3.23E-11 | 2.308063 | 0.021161974 | - | - | Risk |
| rs3129705 | 6 | 30342700 | 9.31E-11 | 1.8275 | 0.033522968 | - | - | Risk |
| rs3130404 | 6 | 30340229 | 5.42E-10 | 1.864301 | 0.016603655 | - | - | Risk |
| rs1793895 | 6 | 31300171 | 2.34E-09 | 2.808737 | 0.00761636 | - | - | Risk |
| rs7382464 | 6 | 33258246 | 5.48E-07 | 2.442648 | 0.012568611 | COL11A2 | Intron | Risk |
| rs209163 | 6 | 28944796 | 6.17E-07 | 1.921266 | 0.029368847 | - | - | Protective |
| rs3131074 | 6 | 29032509 | 7.85E-07 | 1.936084 | 0.046108774 | - | - | Protective |
| rs12193110 | 6 | 30045083 | 2.40E-06 | 2.411796 | 0.004093556 | - | - | Risk |
| rs7398833 | 12 | 110271275 | 2.90E-06 | 2.654621 | 0.012158768 | CUX2 | UTR-3 | Risk |
| rs1150709 | 6 | 28315342 | 1.86E-05 | 2.411646 | 0.009553684 | - | - | Protective |
| rs3893660 | 16 | 11101431 | 3.51E-05 | 2.667258 | 0.024241315 | CLEC16A | Intron | Risk |
| rs2666236 | 10 | 33458878 | 6.78E-05 | 2.331984 | 0.04005377 | - | - | Protective |
| rs3793975 | 11 | 88600836 | 1.39E-04 | 2.780267 | 0.042712233 | TYR | Intron | Risk |
| rs2078851 | 12 | 110174962 | 1.89E-04 | 2.364688 | 0.003118863 | CUX2 | Intron | Risk |
| rs16845023 | 2 | 141433533 | 2.03E-04 | 3.068603 | 0.001263662 | LRP1B | Intron | Protective |
| rs1063588 | 2 | 74543886 | 2.21E-04 | 2.608295 | 0.00467714 | GCS1 | missense | Protective |
| rs807722 | 6 | 24387914 | 4.66E-04 | 3.521206 | 0.002468042 | DCDC2 | Intron | Risk |

**Table S2b – Type 2 Diabetes**

| **SNP** | **Chr** | **Chr Pos** | **Association P-value** | **iHS** | **LRH** | **Gene Symbol** | **Mutation** | **Allele Selected** |
| --- | --- | --- | --- | --- | --- | --- | --- | --- |
| rs7193144 | 16 | 52368187 | 4.78E-08 | 2.11182 | 0.024777165 | FTO | intron | Risk |
| rs13373826 | 1 | 75743383 | 1.28E-07 | 1.881441 | 0.042527714 | SLC44A5 | intron | Protective |
| rs1525791 | 7 | 39123083 | 1.69E-07 | 1.911989 | 0.00327387 | POU6F2 | intron | Risk |
| rs2314349 | 3 | 184573792 | 4.67E-07 | 2.644162 | 0.033413642 | MCF2L2 | intron | Protective |
| rs10149848 | 14 | 71924915 | 2.73E-04 | 2.580795 | 0.024857492 | RGS6 | intron | Protective |
| rs7627205 | 3 | 113917621 | 3.91E-04 | 3.082912 | 0.017947899 | - | - | Protective |
| rs3802678 | 10 | 104105385 | 4.28E-04 | 2.41196 | 0.007784711 | GBF1 | intron | Risk |

**Table S2c – Coronary Artery Disease**

| **SNP** | **Chr** | **Chr Pos** | **Association P-value** | **iHS** | **LRH** | **Gene Symbol** | **Mutation** | **Allele Selected** |
| --- | --- | --- | --- | --- | --- | --- | --- | --- |
| rs6531531 | 4 | 32573012 | 4.09E-10 | 2.777751 | 0.02058147 | - | - | Risk |
| rs7775358 | 6 | 91726062 | 1.45E-06 | 2.039403 | 0.04599021 | - | - | Risk |
| rs10884019 | 10 | 83747001 | 8.01E-05 | 1.74214 | 0.02851118 | NRG3 | Intron | Risk |
| rs7513804 | 1 | 8967711 | 2.11E-04 | 1.87055 | 0.01357487 | - | - | Protective |
| rs6470733 | 8 | 87288188 | 3.06E-04 | 2.365632 | 0.04054805 | - | - | Protective |
| rs9327479 | 5 | 127990740 | 3.43E-04 | 1.985985 | 0.02069978 | - | - | Protective |
| rs7120675 | 11 | 70728329 | 3.95E-04 | 2.742737 | 0.02053245 | - | - | Risk |
| rs3905125 | 1 | 234931082 | 4.58E-04 | 2.580697 | 0.01977487 | ACTN2 | Intron | Risk |

**Table S2d – Crohn’s Disease**

| **SNP** | **Chr** | **Chr Pos** | **Association P-value** | **iHS** | **LRH** | **Gene Symbol** | **Mutation** | **Allele Selected** |
| --- | --- | --- | --- | --- | --- | --- | --- | --- |
| rs1525791 | 7 | 39123083 | 8.46E-09 | 1.911989 | 0.00327387 | POU6F2 | intron | Risk |
| rs2522057 | 5 | 131829846 | 6.86E-07 | 2.382526 | 0.028152149 | - | - | Risk |
| rs2314349 | 3 | 184573792 | 1.18E-06 | 2.644162 | 0.033413642 | MCF2L2 | intron | Protective |
| rs10958116 | 8 | 83704239 | 4.14E-05 | 1.970381 | 0.017881175 | - | - | Protective |
| rs3936503 | 10 | 35589263 | 9.08E-05 | 2.22802 | 0.04761829 | CCNY | intron | Protective |
| rs17045935 | 4 | 114474018 | 1.03E-04 | 2.569997 | 0.035421541 | ANK2 | intron | Protective |
| rs153423 | 5 | 141794129 | 1.42E-04 | 2.093332 | 0.025218831 | - | - | Protective |
| rs7592050 | 2 | 136175697 | 1.64E-04 | 3.052634 | 0.001483083 | R3HDM1 | intron | Protective |
| rs12563946 | 1 | 68465332 | 3.49E-04 | 1.988521 | 0.020516028 | GPR177 | intron | Protective |
| rs4240626 | 8 | 9508470 | 4.11E-04 | 2.219734 | 0.039124501 | TNKS | intron | Protective |
| rs253443 | 5 | 141774089 | 4.31E-04 | 2.137857 | 0.045561118 | - | - | Risk |
| rs13273033 | 8 | 9578103 | 4.85E-04 | 2.148252 | 0.048265519 | TNKS | intron | Protective |

**Table S2e – Bipolar Disorder**

| **SNP** | **Chr** | **Chr Pos** | **Association P-value** | **iHS** | **LRH** | **Gene Symbol** | **Mutation** | **Allele Selected** |
| --- | --- | --- | --- | --- | --- | --- | --- | --- |
| rs1525791 | 7 | 39123083 | 0.000145926 | 1.911989 | 0.00327387 | POU6F2 | intron | Risk |
| rs10829998 | 10 | 133068191 | 0.00016941 | 1.839586 | 0.007432867 | - | - | Risk |
| rs35585 | 16 | 23584612 | 0.000174146 | 1.768149 | 0.008123724 | DCTN5 | intron | Risk |
| rs6827298 | 4 | 83084952 | 0.000370202 | 1.959438 | 0.03489031 | - | - | Protective |
| rs10031202 | 4 | 173865164 | 0.000406138 | 1.945361 | 0.0403045 | GALNTL6 | intron | Protective |
| rs12640371 | 4 | 35344662 | 0.000437063 | 2.199667 | 0.037699673 | - | - | Protective |

Table S2f – Rheumatoid Arthritis

| **SNP** | **Chr** | **Chr Pos** | **Association P-value** | **iHS** | **LRH** | **Gene Symbol** | **Mutation** | **Allele Selected** |
| --- | --- | --- | --- | --- | --- | --- | --- | --- |
| rs707974 | 6 | 31737478 | 6.62E-26 | 1.908233 | 0.036838926 | C6orf47 | nearGene-5 | Risk |
| rs9268878 | 6 | 32539270 | 1.06E-25 | 2.21541 | 0.027676351 | - | - | Protective |
| rs204989 | 6 | 32269830 | 5.33E-11 | 2.212887 | 0.037739195 | GPSM3 | intron | Protective |
| rs2532921 | 6 | 31030549 | 1.76E-08 | 2.309806 | 0.020794988 | - | - | Risk |
| rs4947244 | 6 | 30062343 | 1.79E-08 | 1.890976 | 0.021634692 | HLA-A | intron | Protective |
| rs2517719 | 6 | 30024232 | 3.35E-07 | 2.978616 | 0.000591026 | HLA-A | intron | Risk |
| rs12193110 | 6 | 30045083 | 7.89E-07 | 2.411796 | 0.004093556 | - | - | Risk |
| rs6531531 | 4 | 32573012 | 1.34E-06 | 2.777751 | 0.020581469 | - | - | Risk |
| rs12874151 | 13 | 59763539 | 8.02E-05 | 1.677583 | 0.019995576 | - | - | Risk |
| rs17394081 | 2 | 45546451 | 2.40E-04 | 2.450734 | 0.000433967 | SRBD1 | intron | Protective |
| rs11162943 | 1 | 80391697 | 2.78E-04 | 2.118174 | 0.046193976 | - | - | Risk |
| rs7512762 | 1 | 80368863 | 4.29E-04 | 2.399186 | 0.030226515 | - | - | Risk |
| rs4425122 | 2 | 45603027 | 4.59E-04 | 2.307476 | 0.045078648 | SRBD1 | intron | Protective |
| rs3764342 | 16 | 77024259 | 4.98E-04 | 3.193207 | 0.001613196 | WWOX | intron | Protective |

Table S2g – Hypertension

| **SNP** | **Chr** | **Chr Pos** | **Association P-value** | **iHS** | **LRH** | **Gene Symbol** | **Mutation** | **Allele Selected** |
| --- | --- | --- | --- | --- | --- | --- | --- | --- |
| rs6840033 | 4 | 141448311 | 1.72E-20 | 3.360763 | 0.013930307 | SCOC | intron | Risk |
| rs1570043 | 20 | 22254243 | 4.59E-05 | 1.920591 | 0.023140872 | - | - | Protective |
| rs300916 | 4 | 144493334 | 5.63E-05 | 2.025592 | 0.010613066 | GAB1 | intron | Protective |
| rs2851504 | 7 | 68750942 | 6.29E-05 | 2.198171 | 0.02308313 | AUTS2 | intron | Risk |
| rs210599 | 7 | 68876335 | 8.83E-05 | 2.558023 | 0.004846775 | AUTS2 | intron | Protective |
| rs595681 | 7 | 68828773 | 1.20E-04 | 2.016727 | 0.016120928 | AUTS2 | intron | Protective |
| rs6964734 | 7 | 143355690 | 1.57E-04 | 2.410332 | 0.042334469 | - | - | Risk |
| rs6964415 | 7 | 46209108 | 2.71E-04 | 1.75129 | 0.034619818 | - | - | Protective |
| rs1617402 | 10 | 95157731 | 3.53E-04 | 2.625656 | 0.045278308 | Myof | intron | Protective |
| rs239943 | 19 | 49066556 | 4.57E-04 | 1.946397 | 0.011976815 | - | - | Protective |
