# Supplementary material for: Extreme Evolutionary Disparities Seen in Positive Selection across Seven Complex Diseases
Source: PLoS One. 2010 Aug 17;5(8):e12236. doi: 10.1371/journal.pone.0012236 (PMC2923198; doi:10.1371/journal.pone.0012236)
Supplement: Table S3 — Five of the SNPs listed in Table S2 appear in more than one disease. In particular, rs1525791 appears in four of the seven WTCCC diseases and the risk-associated allele in this SNP shows more selection than the protective allele. This is in contrast to rs204989, where the susceptibility allele for Type 1 Diabetes and the protective allele for Rheumatoid Arthritis are under selection. (0.04 MB DOC) [file pone.0012236.s005.doc]

**Table S3**

| **Gene Symbol** | **-** | **POU6F2** | **GPSM3** | **MCF2L2** | **-** |
| --- | --- | --- | --- | --- | --- |
| ***SNP*** | ***rs12193110*** | ***rs1525791*** | ***rs204989*** | ***rs2314349*** | ***rs6531531*** |
| T1D-Risk | **X** | **X** | **X** |  |  |
| RA-Risk | **X** |  |  |  | **X** |
| T2D-Risk |  | **X** |  |  |  |
| CD-Risk |  | **X** |  |  |  |
| BD-Risk |  | **X** |  |  |  |
| CAD-Risk |  |  |  |  | **X** |
| RA-Protective |  |  | **X** |  |  |
| T2D-Protective |  |  |  | **X** |  |
| CD-Protective |  |  |  | **X** |  |
